# Supplementary material for: Causal Relationship between Aging and Anorexia Nervosa: A White-Matter-Microstructure-Mediated Mendelian Randomization Analysis
Source: Biomedicines. 2024 Aug 16;12(8):1874. doi: 10.3390/biomedicines12081874 (PMC11351342; doi:10.3390/biomedicines12081874)
Supplement: Supplementary file 1 [file biomedicines-12-01874-s001.zip › Supplementary Figure/Figure S4/Singe SNP plot of MR analysis for TL to ubm-b-1852.pdf]

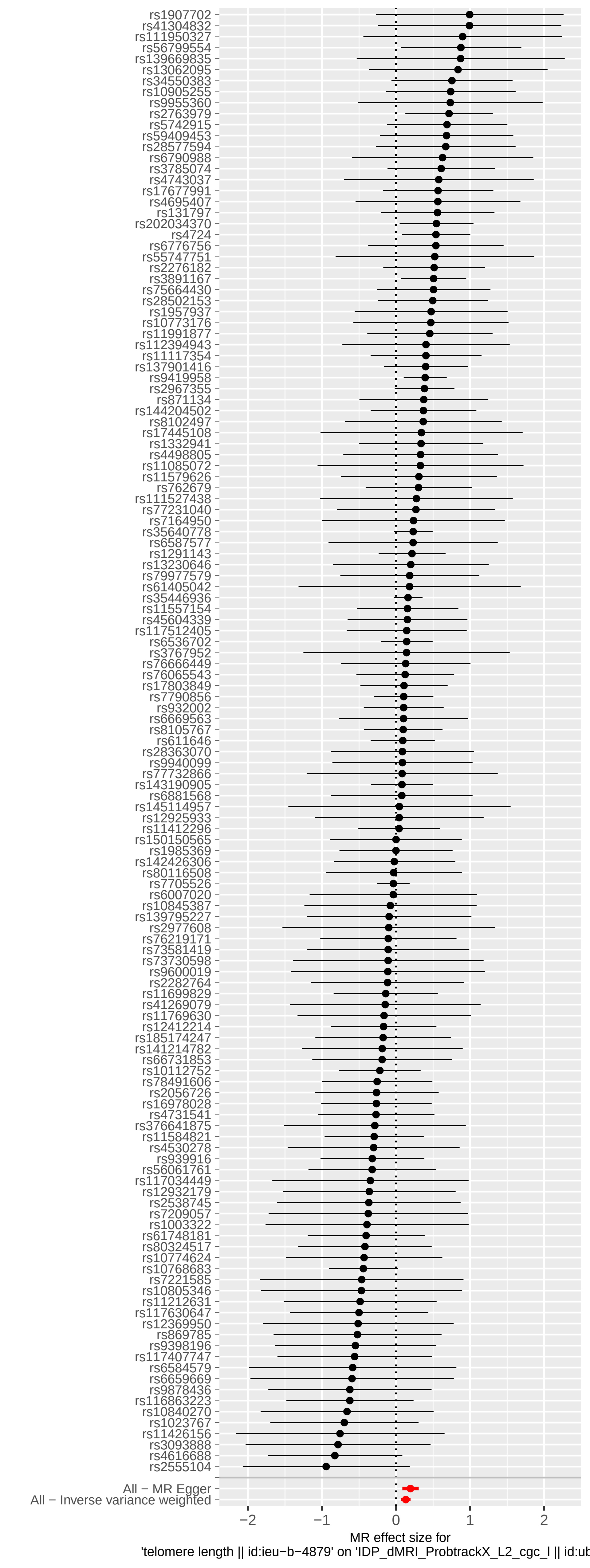

rs1907702
rs41304832
rs111950327
rs56799554
rs139669835
rs13062095
rs34550383
rs10905255
rs9955360
rs2763979
rs5742915
rs59409453
rs28577594
rs6790988
rs3785074
rs4743037
rs17677991
rs4695407
rs131797
rs202034370
rs4724
rs6776756
rs55747751
rs2276182
rs3891167
rs75664430
rs28502153
rs1957937
rs10773176
rs11991877
rs112394943
rs11117354
rs137901416
rs9419958
rs2967355
rs871134
rs144204502
rs8102497
rs17445108
rs1332941
rs4498805
rs11085072
rs11579626
rs762679
rs111527438
rs77231040
rs7164950
rs35640778
rs6587577
rs1291143
rs13230646
rs79977579
rs61405042
rs35446936
rs11557154
rs45604339
rs117512405
rs6536702
rs3767952
rs76666449
rs76065543
rs17803849
rs7790856
rs932002
rs6669563
rs8105767
rs611646
rs28363070
rs9940099
rs77732866
rs143190905
rs6881568
rs145114957
rs12925933
rs11412296
rs150150565
rs1985369
rs142426306
rs80116508
rs7705526
rs6007020
rs10845387
rs139795227
rs2977608
rs76219171
rs73581419
rs73730598
rs9600019
rs2282764
rs11699829
rs41269079
rs11769630
rs12412214
rs185174247
rs141214782
rs66731853
rs10112752
rs78491606
rs2056726
rs16978028
rs4731541
rs376641875
rs11584821
rs4530278
rs939916
rs56061761
rs117034449
rs12932179
rs2538745
rs7209057
rs1003322
rs61748181
rs80324517
rs10774624
rs10768683
rs7221585
rs10805346
rs11212631
rs117630647
rs12369950
rs869785
rs9398196
rs117407747
rs6584579
rs6659669
rs9878436
rs116863223
rs10840270
rs1023767
rs11426156
rs3093888
rs4616688
rs2555104
All − MR Egger
All − Inverse variance weighted
−2
−1
0
1
2
MR effect size for
'telomere length || id:ieu−b−4879' on 'IDP_dMRI_ProbtrackX_L2_cgc_l || id:ub
